# A Novel Mindful-Compassion Art-Based Therapy for Reducing Burnout and Promoting Resilience Among Healthcare Workers: Findings From a Waitlist Randomized Control Trial

**DOI:** 10.3389/fpsyg.2021.744443

**Published:** 2021-10-21

**Authors:** Andy Hau Yan Ho, Geraldine Tan-Ho, Thuy Anh Ngo, Grace Ong, Poh Heng Chong, Dennis Dignadice, Jordan Potash

**Affiliations:** ^1^Action Research for Community Health Laboratory, Psychology Programme, School of Social Sciences, Nanyang Technological University, Singapore, Singapore; ^2^Lee Kong Chian School of Medicine, Nanyang Technological University, Singapore, Singapore; ^3^The Palliative Care Centre for Excellence in Research and Education, Singapore, Singapore; ^4^Assisi Hospice, Singapore, Singapore; ^5^HCA Hospice Care, Singapore, Singapore; ^6^Art Therapy Program, The George Washington University, Ashburn, VA, United States

**Keywords:** burnout, resilience, mindful compassion, art-based therapy, multimodal intervention, palliative end-of-life care, randomized control trial

## Abstract

Protecting the mental health of healthcare workers is an urgent global public health priority. Healthcare workers, especially those immersed in palliative care, are prone to burnout due to the intense emotions associated with end-of-life caregiving. This study examines the efficacy of a novel, multimodal, and group-based Mindful-Compassion Art-based Therapy (MCAT) that integrates reflective self-awareness with creative emotional expression for protecting healthcare workers’ mental health. A dual-arm open-label waitlist randomized controlled trial was conducted. A total of 56 healthcare workers were recruited from the largest homecare hospice in Singapore and randomized to the immediate-treatment condition of a standardized 6-week, 18-hours MCAT intervention (*n*=29), or the waitlist-control condition (*n*=27). Self-administered outcome measures on burnout, resilience, emotional regulation, self-compassion, death attitudes, and quality of life were collected at baseline, post-intervention/second-baseline at 6weeks, and follow-up/post-intervention at 12weeks. Results from mixed model ANOVAs reveal that treatment group participants experienced significant reduction in mental exhaustion, as well as significant improvements in overall emotional regulation, nonreactivity to intrusive thoughts, approach acceptance of death, and afterlife belief as compared to waitlist-control immediately after MCAT completion. Effect sizes of these impacts ranged from medium to large (*η*^2^=0.65 to 0.170). Results from one-way ANOVAs further reveal that the treatment gains of reduced mental exhaustion and increased emotional regulation were maintained among treatment group participants at 12-weeks follow-up compared to baseline, with new benefits identified. These include increased ability to observe and describe one’s experiences, elevated overall self-compassion, greater mindful awareness, enhanced common humanity, and better quality of life. Effect sizes of these impacts were large (*η*^2^=0.128 to 0.298). These findings reflect the robust effectiveness and positive residual effects of MCAT for reducing burnout, building resilience, nurturing compassion, fostering collegial support, and promoting mental wellness among healthcare workers. The clinical model and applicability of MCAT in larger and more diverse caregiving contexts, such as family dementia care, are discussed.

**Clinical Trial Registration:** ClinicalTrials.gov # NCT03440606, #NCT04548089.

## Introduction

Healthcare workers, especially those immersed in palliative care, engage with life and death situations and dilemmas on a day-to-day basis and are exposed to the immense stress of end-of-life caregiving. Amidst these challenges, they are expected to provide unwavering compassionate care to dying patients and their families with immediate responsiveness (Vachon, 1995). However, they receive minimal support for the mental, emotional, and spiritual strains that result from the intense nature of their work, as they often suffer in silence from the vicarious trauma of witnessing the sufferings and deaths of their patients. Repeated studies have found that burnout is a common phenomenon found among healthcare workers caring for dying patients, as recurrent encounters with grief and loss, coupled with a lack of self-care and mounting work-related stress are all conduits to poor mental health (Koh et al., 2015, 2020; Ho, 2021). The negative effects of unresolved work-related stress can trickle down to patients and the rest of the healthcare team, threatening the quality, safety, and integrity of patient care. Despite the many detrimental impacts of burnout, there is a scarcity of holistic and empirically tested interventions to support the mental health of healthcare workers (Mateen and Dorji, 2009). There is clearly an urgent need to develop an effective mental health self-care program for professional careers, as protecting their mental health is an urgent global public health priority (World Health Organization, 2016).

Burnout is a reaction to chronic job-related stress and occurs when individuals become overwhelmed with the mental, emotional, and physical distress associated with their professional work. Defined as “a state of exhaustion in which one is cynical about the value of one’s occupation and doubtful of one’s capacity to perform” (Maslach et al., 1996), burnout can translate into a “literal collapse of the human spirit” (Storlie, 1979), leading to various mental health morbidities including depression, anxiety, and hopelessness (Huang et al., 2009). Such effects inevitably result in loss of clinical hours that amount to major financial losses in healthcare systems (Dill and Cagle, 2010), an example being an estimated US$4.6 billion annual loss in the US healthcare due to such causes (Han et al., 2019). The impact of burnout extends to physical health, correlating with somatic complaints, weakening of the immune system, diabetes, coronary heart disease, cardiovascular diseases, and musculoskeletal pain (Melamed et al., 2006; Salvagioni et al., 2017). Burnout has also been found to cause apathy and hopelessness among healthcare workers, negatively affecting their self-esteem, expression of empathy, and safety of care (Dewa et al., 2017; Wilkinson et al., 2017). Most alarmingly, burnout has consistently been identified as the leading cause of major medical errors among surgeons and physicians in active practice (Shanafelt et al., 2010; Tawfik et al., 2018). Repeated studies have found that healthcare workers around the world are experiencing alarmingly high levels of burnout, ranging from 47 to 70% in the US (Campbell et al., 2010; Ripp et al., 2011), and 71.8 to 80.7% in Singapore (See et al., 2016; Lee et al., 2018). Dill and Cagle (2010) further reported that due to stress and burnout, turnover rate of in-patient hospice workers stands at a worrying 30% and reaches as high as 60% for homecare workers, posing great disruptions to care continuity as well as threats to care quality. These figures may very well worsen with population aging and the relative increase in the demand for palliative care in all advanced societies.

End-of-life caregiving, by its very nature, necessitates strong levels of psycho-socio-emotional competence. Adequately supporting healthcare workers to better cope with caregiving stress requires interventions that promote self-care behaviors for enhancing one’s sense of autonomy, emotional regulation, and empathic capacity (Rushton et al., 2013). Such interventions would need to provide avenues to cultivate resilience *via* meaning-making to derive a renewed appreciation and purpose of one’s caregiving roles (Ablett and Jones, 2007). Of particular, importance is establishing a communal platform for healthcare workers to periodically reflect on their own attitudes, feelings, and anxieties related to loss and grief, while being able to express and share their thoughts with their peers to build mutual respect, compassionate understanding, and collegial support (Chan and Tin, 2012). Emotion-focused and meaning-focused coping strategies that incorporate creativity and expressive arts have proven to best help achieve these intervention goals for empowering healthcare workers (Nainis, 2005).

Over the past two decades, a burgeoning of the literature on mindfulness practice and art-based therapy has revealed their beneficial effects on mental health promotion and stress reduction. Mindfulness practice enables individuals to tune into their immediate experience and emotionality with openness, curiosity, and acceptance (Bishop et al., 2008), thus fostering a deepened understanding of self with greater emotional regulation, together with the potential for developing self-kindness and self-compassion toward painful experiences (Neff, 2003). A 2014 systematic review and meta-analysis of high-quality randomized controlled trials (RCT) found that mindfulness-based interventions provide both short-term and long-term benefits to individuals’ physical and psychological health, including the reduction of stress, anxiety, depressive symptoms, and improvement in chronic disease management (Victorson et al., 2015). Art therapy provides individuals with the means to reframe and communicate experiences and feelings that are difficult to comprehend and verbalize, enabling deep reflections, and creative self-expressions that transcend the barriers of language (McNiff, 2007), thereby empowering one’s sense of self-mastery, interconnectivity, and capacity for healing (Potash et al., 2018). A 2016 systematic review found that art-based therapy is effective in the treatment and aids the recovery of people suffering from depression, post-traumatic stress disorder, and other mental illnesses (Van Lith, 2016). Health and mental health professionals can obtained training and support from qualified art therapists to ethically integrate art making into their clinical practices as a therapeutic tool (Kalmanowitz and Potash, 2009).

The integration of mindfulness practices and art-based therapy for addressing the self-care needs of palliative care professionals is a scarcely explored area (Rappaport, 2013). Building on the clinical foundation of art therapy-based supervision (Potash et al., 2014, 2015) for palliative care workers, while augmenting it with a carefully curated program of mindful-compassion practices (Gilbert and Choden, 2014), together with didactic learning processes that accentuate self-care knowledge through psychoeducation (Brown, 2018), a novel Mindful-Compassion Art-based Therapy (MCAT) for mental health self-care was developed. MCAT is a highly structured, multimodal, and group-based intervention that aims at creating a supportive platform for healthcare workers to deeply reflect, explore, and creatively express their insights and experiences of stress and self-care, caregiving competences and caregiving challenges, loss and grief in facing patients’ death, and aspirations and meaning of caregiving. These interactive processes serve to foster self-understanding, interconnectedness, internal strength, and self-compassion. The ultimate goal of MCAT is to alleviate burnout, cultivate resilience, and promote mental wellness among healthcare workers caring for dying patients. This article reports the clinical efficacy of MCAT. As a pilot study, no *a priori* hypotheses were developed.

## Materials and Methods

This intervention study adopted a dual-arm open-label waitlist RCT design comprising two groups: an immediate-treatment group and a waitlist-control group. The trial was registered on February 22nd, 2018. Healthcare workers were recruited for the study. Pre-, post-, and follow-up data were collected and analyzed to evaluate intervention effectiveness in achieving the stated objectives. Ethical approval was received from the Nanyang Technological University’s Institutional Review Board (IRB-2015-04-021) prior to the commencement of the study.

### Sampling

Study participants comprised 56 frontline healthcare workers recruited from HCA Hospice Care, the largest home hospice care provider in Singapore (*N*=56). Sample size was calculated based an 80% power to detect an effect size (Cohen’s d) of 0.8 (Lambert and Ogles 2004) between the treatment group and the control group at 5% level of significance (two-tailed test); the minimum sample required is 52, or 26 in each group. Inclusion criteria included healthcare workers (i.e., physicians, nurse, medical social workers, and allied health professionals) whose primary job was caring for terminally ill patients, 21years old and above, and fluent in both written and spoken English. Exclusion criteria included the inability to provide informed consent or major depression (or other mental health conditions) or both.

### Intervention Design

Mindful-Compassion Art-based Therapy comprised 6week, 18 hours, standardized, and group-based intervention that integrates the reflective power of mindfulness meditation with the expressive power of art-based therapy to support and enhance the psycho-socio-emotional health of healthcare workers. MCAT was collaboratively delivered by two MCAT therapists including one accredited art therapist and one clinical researcher trained in mindfulness-based stress reduction. Each MCAT group was heterogeneous and included 9–10 physicians, nurses, social workers, allied health professionals, personal care workers, and hospice program staffs. Each MCAT session covered a unique topic that aims to promote understanding, acceptance, and compassion for self and others to cultivate psychological resilience and shared meaning. The topics are strategically designed to build upon each other week by week with a scaffolding framework that deepens the exploration of self-awareness, the practice of self-care, and the promotion of communal support though a series of connected themes Starting with Week 1 – Overview of and Empowering Self-Care; followed by Week 2 – Understanding and Transforming Stress; Week 3 – Inspirational Caregiving; Week 4 – Challenging Caregiving; Week 5 – Understanding Loss and the Impact of Grief; and concluding with Week 6 – Renewing Aspirations and Meaning Reconstruction. Each weekly session began with a check-in session to provide opportunities for participants to discuss questions relevant to the materials covered in previous weeks while allowing MCAT therapists to make a connective transition between weekly topics and themes (5min); this is followed by a mini psychoeducation module comprising of an interactive lecture support by visual aids that introduces the foundational theory and empirical research that accentuate the selected topic (10min); a mindfulness meditation with a theme-based guided imagery exercise (25min); a facilitated expressive art-making session that center on the selected theme (45min); a short break (10min); a creative and reflective writing activity (35min); small group and large group sharing (40min); and ending with a mindful breathing check-out (5min). The integration of these therapeutic elements aimed to provide participants with the foundational knowledge on various aspect of self-care, deepen cognitive awareness, and understanding of their emotionality, while empowering them to be fully aware and articulate of the immediacy of their experiences, and to appreciate the authenticity of their self-reflections, creative expressions, personal insights, and collective wisdoms generated through individual and group work. A detailed intervention protocol is published elsewhere (Ho et al., 2019), and Table 1 outlines the MCAT intervention components.

**Table 1:** Mindful-Compassion Art-based Therapy intervention framework.

Session	Weekly topics & psychoeducation	Mindfulness meditation	Visualization theme	Art making, creative, and reflective writing activities
Week 1	Overview of & Empowering Self-Care	Affectionate Breathing	Self-Kindness	Mandala of Self-Care/Reflective Art Observation
Week 2	Understanding & Transforming Stress	Compassionate Body Scan	Bodily Stress	Symbol of Stress/Transformative Art and Reflective writing
Week 3	Inspirational Caregiving	Loving Kindness Meditation	Strengths in Patient Care	Symbol of Strength/Arts Observation and Creative Response Writing
Week 4	Challenging Caregiving	Loving Kindness Meditation	Challenges in Patient Care	Symbol of Limitation/Art Observation & Creative Response Art Writing
Week 5	Understanding Loss & the Impact of Grief	Meditation on Impermanence	A Patient’s Death	Symbol of Grief/Collective Small Group Mural & Reflective writing
Week 6	Renewing Aspirations & Meaning Reconstruction	Meditation on Giving & Receiving Compassion	Wisdom Learnt & Meaning of Work	Mandala of Meaning/Collective Large Group Mural & Reflective writing

### Research Procedures

**Figure 1:**
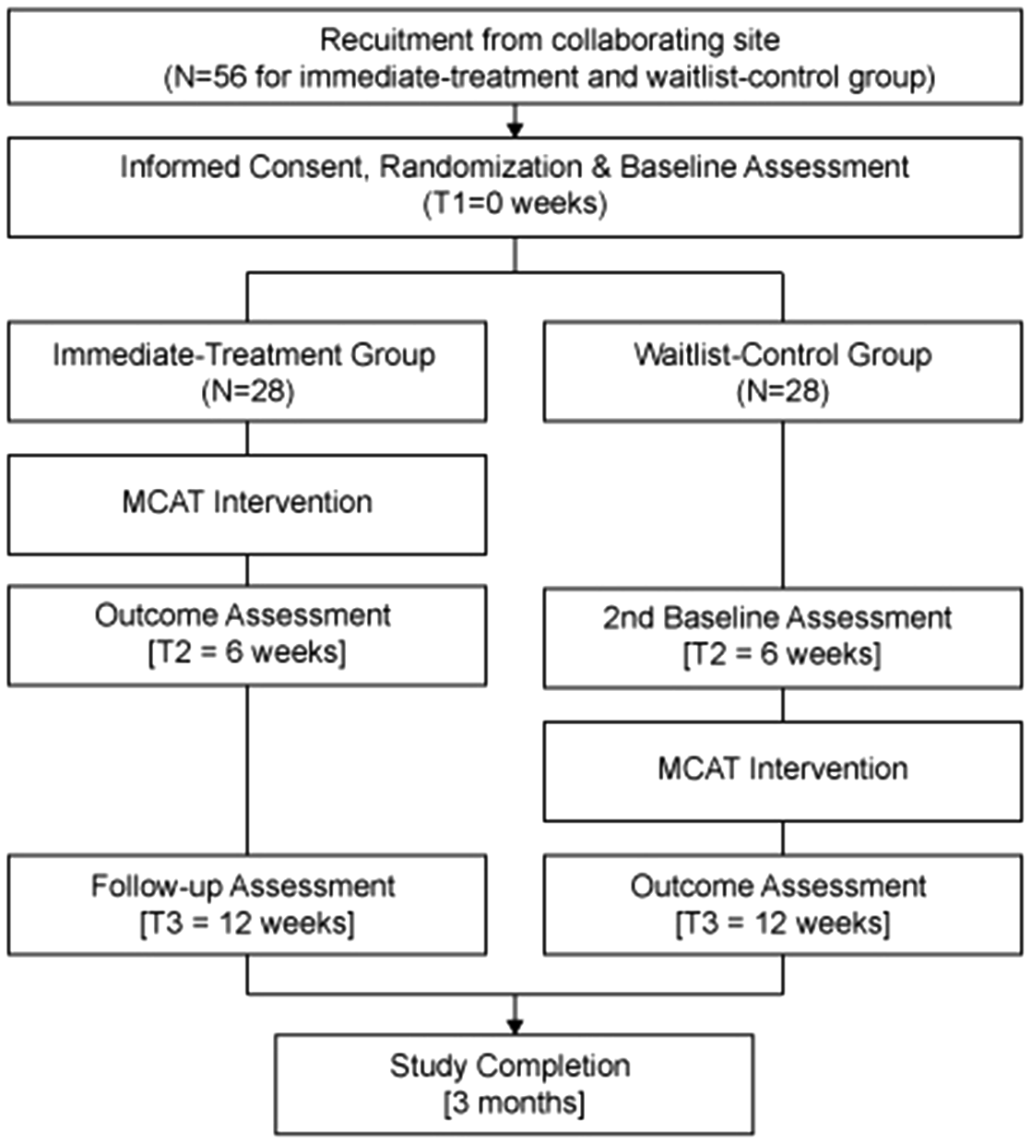
Study Flow Diagram.

Potential participants were referred to the research team by the medical director of collaborating site, with the understanding that they would be given allocated time during regular working hours to participate in the MCAT intervention without any financial implications. Potential participants were also assured that refusal to participate is respected and would not result in any negative consequences. Recruitment was conducted through 3 sequentially overlapping rounds, and each recruitment round comprised 18–20 participants. Upon completion of informed consent and baseline assessments, participants were randomized in either the immediate-treatment group or the waitlist-control group. Simple randomization for each recruitment round was conducted by using an allocation sequence based on a computer-generated list of random numbers. Specifically, a random number sequence ranging from 1 to 18 or 20 (depending on the number of participants recruited in each recruitment round) was generated *via* Research Randomizer (Urbaniak and Plous, 2019). Thereafter, each participant was randomly assigned a unique number from the sequence. Participants whose numbers occupy the first nine to 10 slots in the sequence were assigned to the immediate-treatment group, whereas participants whose numbers occupy the last nine or 10 slots were assigned to the waitlist-control group. Self-administered quantitative assessments were conducted for both groups at baseline (T1), thereafter the immediate-treatment group underwent the 6-week MCAT intervention. Participants in the waitlist-control group did not receive any intervention for the first 6weeks. Upon completion of MCAT among the immediate-treatment group, both groups were assessed again (T2). Subsequently, the waitlist-control group received the same 6-week MCAT intervention. At the end of all intervention components, a final exit assessment was conducted on both groups (T3). A flow diagram of recruitment and study conduct is provided in Figure 1.

### Outcome Measures

Outcomes were assessed with quantitative and qualitative measures. All study participants were assessed by a battery of standardized self-reported psychometric measures on burnout, resilience, emotional regulation, and quality of life at baseline (T1), immediately post-intervention/second-baseline at 6weeks (T2) and follow-up assessment/immediately post-intervention at 12weeks (T3). In addition to the quantitative assessment, large group sharing during from all MCAT sessions were recorded with participants’ consent and transcribed verbatim for analysis.

#### Quantitative Measures

Demographic information including age, gender, marital status, religion, professional roles, employment status, and years of professional experience in end-of-life care was collected at baseline. Primary outcomes included burnout and resilience. Burnout was assessed by the 16-items Maslach Burnout Inventory – General Survey (MBI-GS; Maslach et al., 1996), with higher scores representing greater work-related stress (Baseline Cronbach *α*=0.81). The MBI-GC assesses three domains of burnout, including exhaustion, cynicism, and professional efficacy; scoring for professional efficacy is reversed, with lower scores representing greater burnout. Resilience was assessed by the 11-item Ego-Resilience Revised Scale (ER-11; Farkas and Orosz, 2015), with higher scores corresponding to greater trait resilience (Baseline Cronbach’s *α*=0.78). The ER-11 assesses three domains of resilience, including active engagement with the world, problem-solving strategies, and integrated performance under stress. The MBI-GS and ER-11 possess internal validity, reliability, and cross-cultural applicability.

Secondary outcomes included self-reported levels of emotional regulation, self-compassion, death attitude, and quality of life. Emotional regulation was assessed by the 39-items Five Facet Mindfulness Questionnaire (FFMQ; Baer et al., 2008), with higher scores representing higher level of emotional regulation (Baseline Cronbach *α*=0.89). The FFMQ assesses five domains of emotional regulation, including the ability to observe, and describes one’s experience, act with awareness, non-judgment of experience, and nonreactivity to intrusive thoughts. Self-compassion was assessed by the 12-items Self-Compassion Scale (SCS) Short Form (Raes et al., 2011), with higher scores representing higher self-compassion (Baseline Cronbach *α*=0.84). The SCS assesses six domains of self-compassion, including self-kindness, mindfulness, common humanity, self-judgment, isolation, and over-identification; scoring for self-judgment, isolation, and over-identification are reversed, with lower score indicating higher self-compassion. Death attitude was assessed by the 32-items Death Attitude Profile-Revised (DAP-R; Wong et al., 1994), which measures seven unique domains of death attitudes, with higher scores representing higher levels of fear of death (Baseline Cronbach *α*=0.85), death avoidance (Baseline Cronbach *α*=0.90), approach acceptance (Baseline Cronbach *α*=0.81), escape acceptance (Baseline Cronbach *α*=0.91), neutral acceptance (Baseline Cronbach *α*=0.82), personal acceptance (Baseline Cronbach *α*=0.62), and afterlife belief (Baseline Cronbach *α*=0.88; Ho et al., 2010). Finally, quality of life was measured by the 8-item EUROHIS Quality of Life Scale-8 (EUROHIS-QoL-8; da Rocha et al., 2012), with higher scores representing greater quality of life (Baseline Cronbach’s *α*=0.84). Again, the FFMQ, SCS, DAP-R, and EUROHIS-QoL-8 possess internal validity, reliability, and cross-cultural applicability.

#### Qualitative Measures

Weekly group sharing was audio recorded and transcribed verbatim. In addition, all artworks together with creative and reflective writings created by study participants were documented and categorized as supplementary data. To protect the confidentiality of the participants, identifying information was removed and pseudonames were assigned to each participant before analysis.

### Data Analyses

Quantitative data were entered, stored, and analyzed using SPSS statistical analysis software. Baseline demographic characteristics between intervention and waitlist-control groups are presented either number (%) for categorical variables or mean (SD: standard deviation) for quantitative variables. The immediate-treatment group and waitlist-control group were compared on the primary outcomes and secondary outcomes. To examine the changes in continuous outcome variables between group and over, mixed model ANOVAs were conducted for each outcome with the appropriate means, F ratio, *value of p*, and the effect size estimates of Eta-Squared (*η*^2^) reported. *Post-hoc* tests using the Bonferroni correction were also conducted to control for error. Moreover, one-way ANOVAs were performed for the immediate-treatment group with an additional time point. All analysis was adjusted for baseline demographic variables (age, gender, marital status, education, religion, employment status, professional roles, and years of professional experience). All *p*-values were based on two-tailed tests of significance and those less than 0.05 were considered statistically significant. Qualitative data were managed by the QSR NVIVO software package. Weekly group sharing was audio recorded, transcribed verbatim, and verified by research team members, as well as all participants’ creative and reflective writings. All qualitative data were analysis using thematic analysis which involved several steps of data reduction and data reconstruction (Braun and Clarke, 2006). First, authors 1 and 2 conducted multiple reading of the transcripts and narrative writings to familiarized themselves with the data. Author 1 and 2 then conducted line-by-line coding to develop descriptive themes and analytical categories that represented a patterned response or meaning within the data which captured something important in relation to the research question (Pope et al., 2000). This was followed by regular meetings among all authors for the further refinement of themes and categories to encapsulate the meaning and content within the cluster of similar codes, with the emergent themes and sub-themes created *via* a summary chart. All authors reviewed and defined the emergent themes; once consensus was reached, and operational definitions were created. To maximize credibility, criticality, and authenticity, strategies, such as theory triangulation, research triangulation, and member checking, were exercised throughout the analytical process.

## Results

### Participant Demographics

A total of 56 participants were successfully recruited and completed the study with no attrition throughout the entire research period. Participants were aged between 23 to 64years (*M*=44.40, *SD*=10.97), predominantly female (75%) and have completed a bachelor’s degree or above (77%). The majority of participants was nurses (48%), followed by physicians (14%), medical social workers (14%), and allied care professionals (14%). The years of end-of-life care experience ranged from l to 30years (*M*=5.08, *SD*=5.80), with the majority having 1 to 5years of experience (70%). There were no statistically significant differences in demographic measures between treatment group and control group. Please refer to Table 2 for more information regarding participants’ demographics.

**Table 2:** Participant demographic information.

Demographic characteristic	Immediate treatment	Waitlist control
	(*n*=29)	(*n*=27)
	Mean (*SD*) or *N* (%) or Range	
**Age in years, Mean (*SD*)**	43.52 (11.54)	45.27 (10.39)
Range	23 to 65years	28 to 61years
**Gender (Female)**	22 (75.8614)	20 (74.07)
**Marital Status**		
Single/Divorce/Widowed	11 (37.93)	15 (59.26)
Married	18 (62.07)	12 (44.84)
**Education**		
High School/College	6 (20.7)	7 (25.9)
Bachelor’s degree	17 (58.6)	14 (40.7)
Master’s degree	4 (13.8)	8 (29.6)
Doctorate’s degree	2 (6.9)	1 (3.7)
**Religion**		
Catholic	6 (20.69)	0
Christian	11 (37.93)	13 (48.15)
Buddhist	5 (17.24)	5 (18.52)
Taoist	2 (6.90)	1 (3.70)
Muslim	1 (3.45)	1 (3.70)
Hinduism	4 (13.79)	4 (14.81)
No religion	0	3 (11.11)
**Professional Roles**		
Physicians	5 (17.24)	3 (11.11)
Nurses	17 (58.62)	10 (37.04)
Medical Social Workers	3 (10.34)	5 (18.52)
Personal Care Workers	2 (6.9)	3 (11.11)
Allied Health Workers	2 (6.9)	6 (22.22)
**Years of Experience in EoL Care (*SD*)**	5.63 (5.86)	4.44 (5.52)
Range	1 to 30years	1 to 25years
1 to 5years	20 (68.96)	19 (70.37)
6 to 10years	7 (24.14)	6 (22.22)
10years of above	2 (6.88)	2 (7.41)

### Quantitative Findings

Results from mixed model ANOVAs reveal significant interaction effects between immediate-treatment group and waitlist-control group across time. Specially, treatment group participants experienced significant reduction in mental exhaustion [16.48 vs. 17.48; *F*(2, 108)=3.27, *p*=0.042, *η*^2^=0.065] immediately upon MCAT completion as compared to waitlist control. Treatment group participants also experienced significant improvements in overall emotional regulation [16.89 vs. 16.37; *F*(1.7, 91.8)=5.34, *p*=0.006. *η*^2^=0.170], nonreactivity to intrusive thoughts [3.46 vs. 3.29; *F*(1.7, 91.8)=5.32, *p*=0.009, *η*^2^=0.090], approach acceptance of death [42.03 vs. 39.07; *F*(1.7, 93.7)=4.22, *p*=0.022, *η*^2^=0.072], and afterlife belief [11.21 vs. 9.78; *F*(2, 108)=3.97, *p*=0.022, *η*^2^=0.068] immediately after intervention as compared to waitlist control. Effect sizes of these changes were medium to large. These findings reflect MCAT’s robust efficacy for burnout reduction and wellness promotion among healthcare workers. Details of mixed model ANOVAs are provided in Table 3.

**Table 3:** Outcome comparisons between treatment and control groups using mixed model ANOVAs.

Variables	Immediate treatment		Waitlist control		Group			Time			Group x Time		
	(*N*=29)		(*N*=27)		Effect			Effect			Interaction		
	*T1*	*T2*	*T1*	*T2*	*F ratio*	*df*	*η* ^2^	*F ratio*	*df*	*η* ^2^	*F ratio*	*df*	*η* ^2^
	*Means (SD)*	*Means (SD)*	*Means (SD)*	*Means (SD)*									
**Primary Outcomes**													
Burnout (MBI-GS)	49.1 (12.04)	45.83 (11.66)	46.37 (12.73)	46.22 (13.11)	0.11	1	0.002	3.27^*^	2	0.057	1.15	2	0.021
Exhaustion	20.79 (6.59)	16.48 (5.79)	17.78 (5286)	17.48 (6.05)	0.52	1	0.010	9.13^***^	2	0.145	3.76^*^	2	0.065
Cynicism	12.83 (6.44)	12.69 (5.29)	13.96 (5.19)	13.11 (4.54)	0.51	1	0.009	0.59^a^	1.66	0.011	0.13^a^	1.66	0.002
Professional Efficacy	32.52 (6.44)	32.48 (5.29)	33.37 (5.19)	33.22 (4.54)	0.61	1	0.011	0.01	1.58	<0.001	0.25^a^	1.58	0.005
Resilience (ERS-11)	39.34 (5.518)	41.52 (5.67)	42.30 (5.34)	42.26 (5.69)	2.40	1	0.043	2.95^†^	1.8	0.052	2.96^†^	1.8	0.052
Active Engagement	13.48 (3.23)	14.48 (3.07)	14.48 (2.56)	14.33 (2.87)	0.65	1	0.012	1.66	2	0.030	2.52	2	0.045
Performance under Stress	5.69 (1.34)	6.00 (1.31)	6.22 (1.09)	6.15 (1.07)	1.77	1	0.032	0.68	2	0.012	0.93	2	0.017
Problem Solving	10.93 (2.05)	11.55 (2.21)	12.04 (1.97)	12.11 (1.89)	3.75^†^	1	0.065	2.84	2	0.050	1.29	2	0.023
**Secondary Outcomes**													
Emotional Regulation (FFMQ)	15.85 (2.36)	16.89 (2.34)	16.43 (1.95)	16.37 (2.19)	<0.001	1	<0.001	11.03^***^	2	0.170	5.34^**^	2	0.170
Observing	3.29 (0.72)	3.57 (0.75)	3.28 (0.69)	3.25 (0.88)	0.91	1	0.016	3.99^*^	2	0.069	2.18	2	0.039
Describing	3.00 (0.66)	3.25 (0.64)	3.36 (0.67)	3.45 (0.79)	1.85	1	0.033	8.24^***^	2	0.132	1.68	2	0.030
Acting with Awareness	3.41 (0.69)	3.53 (0.56)	3.56 (0.72)	3.53 (0.61)	0.03	1	0.001	0.44	1.78	0.008	1.40	1.78	0.025
Nonjudging	2.90 (0.66)	3.08 (0.72)	2.90 (0.71)	2.85 (0.77)	0.84	1	0.015	2.79^†^	2	0.049	1.19	2	0.022
Nonreactivity	3.25 (0.65)	3.46 (0.58)	3.33 (0.62)	3.29 (0.66)	0.12	1	0.002	1.19	1.70	0.022	5.32^a**^	1.70	0.090
Self-Compassion (SCS)	38.45 (7.51)	42.24 (8.09)	39.26 (6.41)	47.78 (6.09)	<0.001	1	<0.001	14.29^***^	2	0.209	0.61	2	0.011
Self-Kindness	7.14 (1.81)	7.75 (1.53)	7.26 (1.40)	7.22 (1.53)	0.10	1	0.002	0.93	2	0.017	1.51	2	0.027
Self-Judgment	5.86 (1.87)	5.17 (1.96)	6.11 (1.39)	5.70 (1.49)	0.76	1	0.014	2.84^†^	2	0.050	0.32	2	0.006
Common Humanity	6.59 (1.74)	7.55 (1.70)	6.96 (1.82)	7.52 (1.37)	<0.001	1	<0.001	7.20^**^	1.77	0.118	1.00^a^	1.77	0.018
Isolation	6.03 (1.52)	5.76 (1.85)	5.56 (1.55)	5.00 (1.84)	1.86	1	0.033	2.02	2	0.036	0.87	2	0.016
Mindfulness	7.24 (1.48)	7.83 (1.37)	7.44 (1.37)	7.93 (1.27)	0.28	1	0.005	4.78^**^	2	0.081	0.042	2	0.001
Overidentified	6.62 (1.80)	5.97 (1.61)	6.70 (1.79)	6.19 (1.73)	0.26	1	0.005	7.57^**^	2	0.123	0.10	2	0.002
Death Attitude (DAP-R)													
Fear of Death	22.41 (8.99)	20.48 (6.78)	21.07 (9.37)	21.00 (8.52)	0.001	1	<0.001	1.84	2	0.033	0.88	2	0.016
Death Avoidance	11.93 (7.16)	11.14 (6.17)	11.04 (5.15)	11.85 (5.27)	0.08	1	0.001	0.02	1.8	<0.001	1.99	1.8	0.035
Neutral Acceptance	19.52 (2.34)	19.59 (2.11)	19.11 (3.14)	19.93 (1.77)	0.22	1	0.004	1.80	1.7	0.032	1.88	1.7	0.034
Approach Acceptance	38.97 (10.55)	42.03 (10.51)	38.41 (13.46)	39.07 (13.94)	0.02	1	<0.001	3.06^†^	1.7	0.054	4.22^*^	1.7	0.072
Escape Acceptance	22.21 (6.47)	22.41 (7.69)	21.37 (8.82)	21.81 (9.43)	0.266	1	0.005	2.25	2	0.040	0.38	2	0.007
Personal Acceptance	9.90 (3.07)	10.45 (3.08)	10.48 (2.88)	10.78 (3.40)	0.51	1	0.009	0.062	2	0.011	0.08	2	0.001
Afterlife Belief	10.41 (3.47)	11.21 (2.85)	10.04 (3.04)	9.78 (3.53)	0.43	1	0.008	0.47	2	0.009	3.97^*^	2	0.068
Quality of Life (EURO-QOL-8)	28.79 (4.69)	31.07 (3.92)	30.74 (4.37)	31.74 (3.10)	0.67	1	0.012	14.51^***^	2	0.212	2.98^†^	2	0.052

* *p*<0.05;

** *p*<0.01 and

*** *p*<0.001.

† Partial significance.

a Greenhouse-Geisser correction was used when the data violated the assumption of sphericity.

Results from follow-up one-way ANOVAs reveal that the treatment gains of reduced mental exhaustion [16.76 vs. 20.97; *F*(2, 56)=13.72, *p*<0.001, *η*^2^=0.329] and increased overall emotional regulation [16.98 vs. 15.85; *F*(2, 56)=12.65, *p*<0.001, *η*^2^=0.311] were maintained among treatment group participants at 12-weeks follow-up compared to baseline, with new benefits identified. These include increased ability to observe [3.59 vs. 3.29; *F*(2, 56)=6.09, *p*=0.004; *η*^2^=0.179] and describe [3.34 vs. 3.00; *F*(2, 56)=9.82, *p*<0.001, *η*^2^=0.260] one’s internal and external experiences, elevated overall self-compassion [41.66 vs. 38.45; *F*(2, 56)=10.80, *p*<0.001, *η^2^*=0.278], greater mindful awareness [7.69 vs. 7.24; *F*(2, 56)=4.10, *p*=0.022, *η*^2^=0.211], enhanced common humanity (or interconnectedness to others) [7.59 vs. 6.59; *F*(2, 56)=7.48, *p*=0.001, *η*^2^=0.128], and better quality of life [32.31 vs. 28.79; *F*(2, 56)=11.89, *p*<0.001, *η*^2^=0.298] among treatment group participant at 12-weeks follow-up compared to baseline. Effect sizes of these changes were large. These findings reflect the positive residual effects of MCAT, as well as its ability to generate new treatment benefits beyond intervention completion. Details of one-way ANOVAs are provided in Table 4.

**Table 4:** Within treatment group analysis using one-way ANOVAs.

**Variables**	**T1**	**T2**	**T3**	**ANOVA**		**T1 vs. T2**		**T1 vs. T3**	
	**Means (*SD*)**	**Means (*SD*)**	**Means (*SD*)**	***F ratio***	***η*** ^**2**^	**95% Confidence**	**Difference**	**95% Confidence**	**Difference**
						**interval**	**(T2 – T1)**	**interval**	**(T3 – T1)**
**Treatment Group (N=29)**									
**Primary outcomes**									
Burnout (MBI-GS)	49.10 (12.056)	45.83 (11.66)	45.48 (12.80)	3.37	0.107	(−6.68, 0.13)	−3.276^†^	(−7.94, 0.70)	−3.621
Exhaustion	20.79 (6.59)	16.48 (5.79)	16.76 (5.44)	13.72^***^	0.329	(−5.64, −0.98)	−3.310^**^	(−5.72, −2.35)	−4.034^***^
Cynicism	12.83 (6.44)	12.69 (5.30)	12.24 (4.83)	0.16	0.006	(−3.53, 3.26)	−0.138	(−3.50, 2.33)	−0.586
Professional Efficacy	32.52 (5.42)	32.48 (5.87)	32.10 (7.28)	0.07	0.003	(−2.14, 2.07)	−0.034	(−4.16, 3.33)	−0.414
Resilience (ERS-11)	39.34 (5.52)	41.52 (5.67)	40.48 (6.74)	3.84^a^	0.121	(0.56, 3.78)	2.172^**^	(−1.32, 3.60)	1.138
Active Engagement	13.48 (3.23)	14.48 (3.07)	14.00 (3.36)	3.12	0.100	(−0.01, 2.01)	1.000^†^	(−0.67, 1.71)	0.517
Performance under Stress	5.69 (1.34)	6.00 (1.31)	5.93 (1.10)	1.23	0.042	(−0.23, 0.85)	0.310	(−0.32, 0.80)	0.241
Problem Solving	10.93 (2.05)	11.55 (2.21)	11.38 (2.19)	1.89	0.063	(−0.24, 1.48)	0.621	(−0.52, 1.42)	0.448
**Secondary outcomes**									
Emotional Regulation (FFMQ)	15.85 (2.36)	16.89 (2.34)	16.98 (2.19)	12.65^***^	0.311	(0.47, 1.61)	1.039^***^	(0.40, 1.86)	1.126^**^
Observing	3.29 (0.72)	3.57 (0.75)	3.59 (0.72)	6.09^**^	0.179	(0.03, 0.52)	0.276^*^	(0.03, 0.58)	0.302^*^
Describing	3.00 (0.66)	3.25 (0.64)	3.34 (0.66)	9.82^***^	0.260	(0.06, 0.44)	0.250^**^	(0.12, 0.56)	0.341^**^
Acting with Awareness	3.41 (0.69)	3.53 (0.57)	3.58 (0.53)	1.74^a^	0.059	(−0.10, 0.35)	0.125	(−0.12, 0.47)	0.172
Nonjudging	2.90 (0.66)	3.08 (0.72)	3.20 (0.71)	4.10	0.128	(−0.04, 0.40)	0.181	(0.02, 0.59)	0.306^*^
Nonreactivity	3.25 (0.66)	3.46 (0.58)	3.26 (0.59)	4.56^a^	0.140	(−0.01, 0.42)	0.207^†^	(−0.23, 0.24)	0.005
Self-Compassion (SCS)	38.45 (7.51)	42.24 (8.09)	41.66 (6.19)	10.80^***^	0.278	(1.26, 6.33)	3.793^**^	(1.11, 5.30)	3.207^**^
Self-Kindness	7.14 (1.81)	7.76 (1.53)	7.31 (1.65)	2.43	0.080	(−0.18, 1.42)	0.621	(−0.61, 0.95)	0.172
Self-Judgment	5.86 (1.87)	5.17 (1.97)	5.76 (1.33)	2.55	0.084	(−1.59, 0.21)	−0.690	(−0.91, 0.70)	−0.103
Common Humanity	6.59 (1.74)	7.55 (1.70)	7.59 (1.43)	7.48^**^	0.211	(0.16, 1.77)	0.966^*^	(0.18, 1.82)	1.000^*^
Isolation	6.03 (1.52)	5.76 (1.85)	5.48 (1.43)	1.78	0.060	(−1.14, 0.59)	−0.276	(−1.22, 0.11)	−0.552
Mindfulness	7.24 (1.48)	7.83 (1.37)	7.69 (1.39)	4.10^*^	0.128	(0.07, 1.10)	0.586^*^	(−0.11, 1.01)	0.448
Overidentified	6.62 (1.80)	5.97 (1.61)	5.69 (1.49)	4.49	0.138	(−1.46, 0.15)	−0.655	(−1.88, 0.14)	−0.931^†^
Death Attitude (DAP-R)									
Fear of Death	22.41 (8.99)	20.48 (6.78)	19.86 (7.40)	2.63	0.086	(−5.06, 1.20)	−1.931	(−5.47, 0.37)	−2.552
Death Anxiety	11.93 (7.16)	11.14 (6.17)	10.86 (5.95)	1.01	0.035	(−2.63, 1.04)	−0.793	(−3.16, 1.02)	−1.069
Neutral Acceptance	19.52 (2.34)	19.59 (2.11)	18.79 (2.56)	1.96^a^	0.065	(−0.72, 0.86)	0.069	(−2.04, 0.60)	−0.724
Approach Acceptance	38.97 (10.55)	42.03 (10.51)	39.52 (11.58)	6.83^**^	0.196	(0.82, 5.32)	3.069^*^	(−1.87, 2.97)	0.552
Escape Acceptance	22.21 (6.48)	22.41 (7.69)	24.17 (8.38)	2.79	0.091	(−2.11, 2.53)	0.207	(−0.62, 4.55)	1.966
Personal Acceptance	9.90 (3.07)	10.45 (3.08)	10.17 (3.21)	0.49	0.017	(−0.97, 2.08)	0.552	(−1.69, 1.64)	0.276
Afterlife Belief	10.41 (3.47)	11.21 (2.85)	10.24 (3.29)	3.49^*^	0.111	(−0.34, 1.93)	0.793	(−1.16, 0.82)	−0.172
Quality of Life (EURO-QOL-8)	28.79 (4.69)	31.07 (3.92)	32.31 (4.36)	11.89^***^	0.298	(0.24, 4.31)	2.276^*^	(1.62, 5.42)	3.517^***^

* *p*<0.05;

** *p*<0.01 and

*** *p*<0.001.

† Partial significance.

a Greenhouse-Geisser correction was used when the data violated the assumption of sphericity.

### Qualitative Findings

The qualitative data from the group sharing among MCAT participants, together with their creative and reflective writing supported by their art works, provided further insights on the intervention’s efficacy and therapeutic mechanisms in reducing burnout, building resilience, nurturing compassion, and fostering collegial support. The following section provides an illustrative summary of the qualitative findings generated through thematic analysis.

#### Reducing Burnout

**Figure 2:**
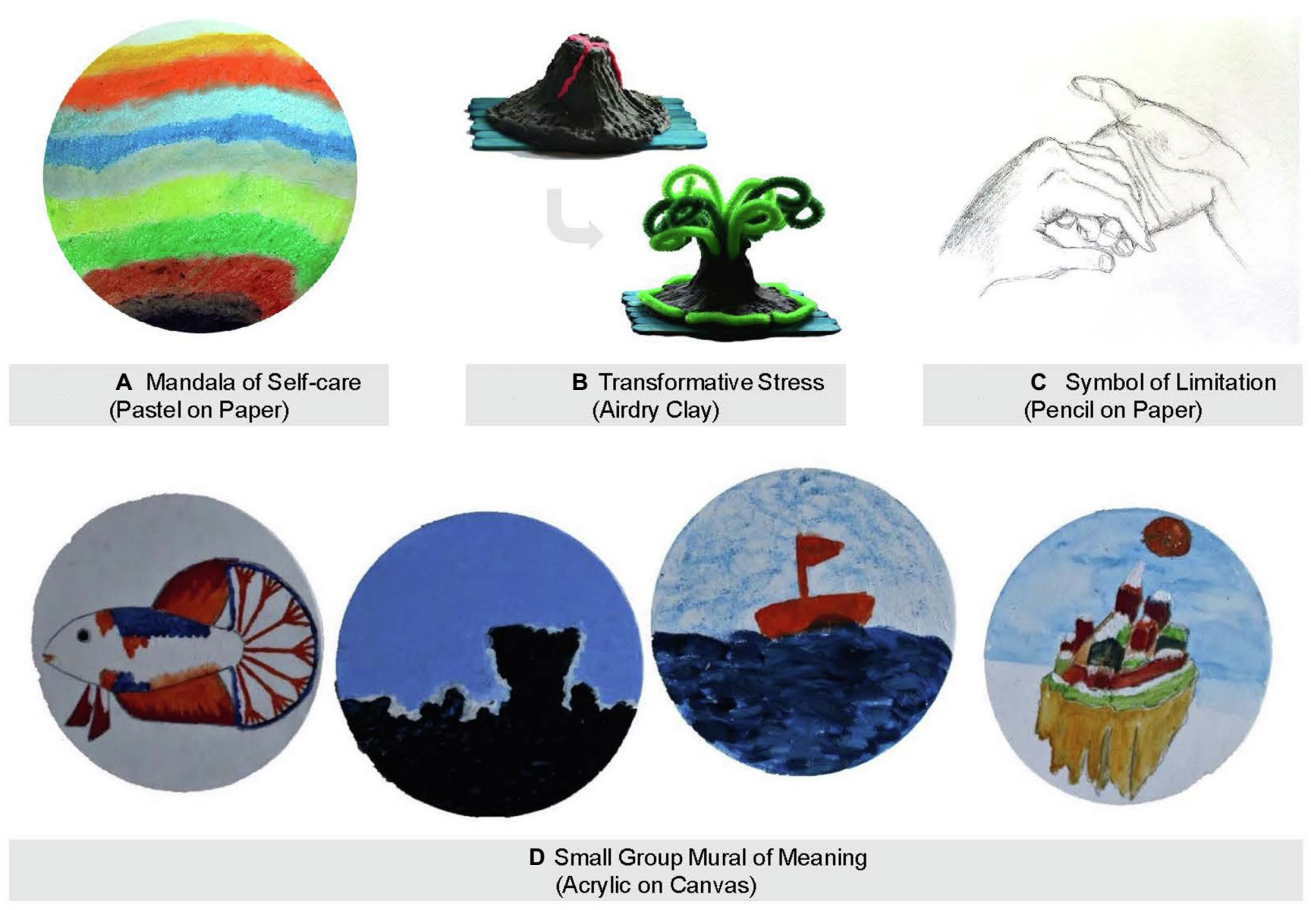
**(A)** Mandala of self-care (Pastel on paper). **(B)** Transformative stress (Airdry clay). **(C)** Symbols of limitation (Pencil on paper). **(D)** Small group mural of meaning (Acrylic on canvas).

The multimodal therapeutic nature of MCAT provided participants with the much-needed time and space to reflect on their own self-care needs and what they can do to alleviate work-related stress. For instance, a 54-year-old medical social worker shared that “*You are expected to fill up quite a lot of shoes… and that’s why it’s so important to put yourself first you know, and care for yourself first.”* Such self-reflection led to a deepened self-understanding and a renewed appreciation for mindful living for calming the emotional burden of end-of-life caregiving. A 47-year-old physician shared *“Our work is filled with strong emotions… Sometimes we really have to pause and just breathe, breathe in peace, calmness and awareness, before we can perform our task again.”* Upon deep reflection followed by the creation of a Mandala of Self-Care and reflective art observation (see Figure 2A), a 36-year-old nurse described in her reflective writing that caring for oneself can be as simple as paying attention to one’s emotion in the immediacy of one’s experience: *“We need to live not in the past nor the future, but in the present.”* These vivid art-based narratives highlight the efficacy of MCAT in burnout reduction and the promotion of mental health self-care.

#### Building Resilience

Understanding one’s sources of stress, illustrating this profound feeling *via* expressive arts, and thereafter articulating the experience through words empowered participants to build internal strengths and resources for coping with work-related stress. A 32-year-old social worker shared *“I wanted to be able to embrace my stress instead of fixing my stress… I feel now (after reflective art-making) I can actually cope with stress better.”* This augmentation of reflective self-awareness with creative self-expression rendered through the MCAT integrative model also enabled participants to look at their experience of loss and grief with greater acceptance and psychological flexibility. A 42-year-old nurse wrote about her experience with losing a patient, *“When all is drowning and sinking in adversity, we need to be still and persevere, to embrace the trapping moves and see them as a dance of life.”* Being able to transform one’s cognitive appraisal of adverse events through creating a Symbol of Stress as represented by an active volcano and thereafter changing it into a lush forest through the transformative art exercises (see Figure 2B), a 28-year-old medical social worker expressed in her reflective writing, “*Stress may not be a bad thing, it can bring out the brilliance in people.”* MCAT’s capability and mechanisms for resilience building are accentuated by the arts and narratives of all study participants.

#### Nurturing Compassion

Mindfulness practices coupled with theme-based guided imagery rendered through MCAT provided a nurturing platform for participants to develop greater empathy and kindness for themselves and for their patients. A 54-year-old nurse shared “*When I am focusing on breathing through my nose… it made me think about my patient who was really struggling for a breath… I came to appreciate the gift of breathing.”* Participants were able to integrate their own experiences with that of their patients – this sparked an awareness and acknowledgment of the interconnectedness and common humanity between them and their patients. A 58-year-old nurse shared *“The journeys of illness are long and waving, there are many ups and downs… many patients struggle to find peace and hope… I wish for them to be safe and calm.”* In creating a Symbol of Limitation that illustrates the challenge and stagnation one had experienced in support dying patients (see Figure 2C), a 53-year-old physician shared in his reflective writing, *“While I want to hold on to the hands of the vulnerable, I also recognize that I myself am a vulnerable being who needs love and support.”* Such eloquent and honest narratives underscore MCAT’s capacity in not only nurturing compassion for others, but also compassion for self.

#### Fostering Collegial Support

**Figure 3:**
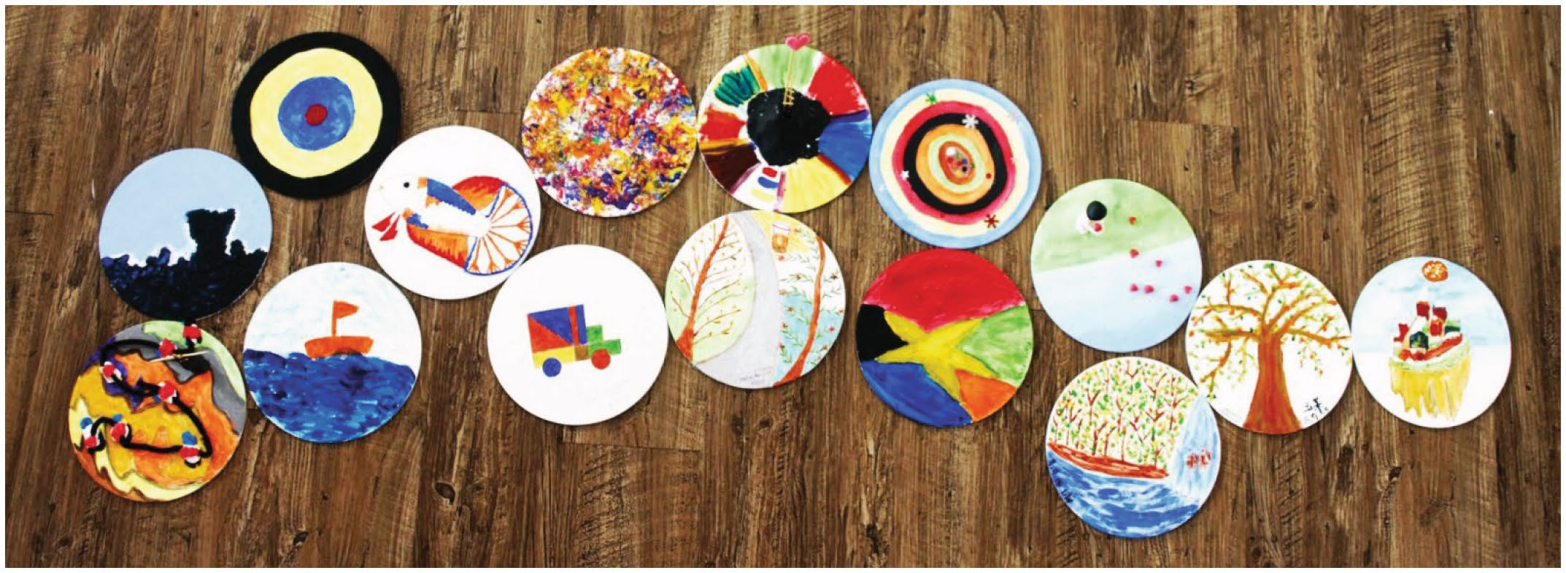
Large group mural – ‘Seasons of Life’ (Acrylic on canvas).

The weekly scaffolding of deepened self-reflection, art making, and group sharing empowered participants to tell their own unique stories as an end-of-life caregiver, while seeing the connection between their own experiences with those of their group members. This mechanism allowed participants to take a bird’s eye view of their collective challenges while finding ways to attain better mental health. A 60-year-old nurse shared *“I see happiness in all the art pieces that were created today… Happiness can be very simple, happiness in what we are content with.”* The emotional connections and relational bonds created were further solidified through the group mural activities in the last 2weeks of MCAT, where participants were asked to identify art pieces that are similar in colors and compositions for developing a joint story that tells their collective experiences. In creating a small group mural on the theme of meaning (see Figure 2D), participants were able to expand their views on life with a more positive and fluid mindset, as they expressed in their reflective writing, *“We are often fixated on looking at things at horizon levels, but what we see is not all that there is. Deep down in the blue see lies beautiful fishes, high up in the heavens there is a beautiful paradise. It is up to us to find meaning of it all.”* In being able to establish a renewed sense of collective meaning, participants widened their perspectives toward life and death with less rigid attachments for greater mental wellness. A large group mural created jointly by all participants of an MCAT group together with the therapists (see Figure 3) were illustrated by their poem entitled “Seasons of Life,” *“Let the fallen leaves be the nourishment for next spring, together in this journey of life.”*

## Discussion

This is the first known empirical study that has developed and tested a multimodal intervention that integrates mindfulness practice and art-based therapy for protecting and supporting the mental health of healthcare workers. MCAT aims to mitigate the detrimental effects of burnout and to foster psychological resilience among the healthcare workers immersed in the field of palliative end-of-life care. Utilizing a robust waitlist RCT design, the overall quantitative findings revealed that MCAT was effective in reducing mental exhaustion, enhancing emotional regulation and nonreactivity to intrusive thoughts, and fostering positive death attitudes among immediate-treatment group participants when compared to waitlist control across time. Treatments gains on mental exhaustion and emotional regulation were maintained at 12-weeks follow-up with new benefits identified, such as improvements in the ability to observe and describe one’s feelings, thoughts and emotions, practice self-compassion and mindful awareness, and experience a deepened sense of common humanity and elevated quality of life. All significant findings are marked by medium to mostly large effect sizes, reflecting the clinical efficacy and positive residual effects of the intervention. The qualitative data and vivid art-based narratives created by study participants also provided valuable insights into MCAT’s therapeutic mechanisms for reducing burnout, building resilience, nurturing compassion, and fostering collegial support among healthcare workers. These findings add robust evidence to the growing literature of mindfulness practice and art-based therapy for health and wellness promotion, together with a novel, integrative, and evidence-based therapeutic modality for mental health self-care.

### Interpreting the Findings

MCAT is founded upon a multimodal paradigm that amalgamates mindful contemplative reflections with art-based self-expressions as well as brief psychoeducation for instilling cognitive, emotional, and behavioral changes that gear toward healing and psychological wellbeing. The striking findings generated from this RCT can be attributed to the various therapeutic underpinnings of this innovative intervention. First, MCAT which is rooted in the foundation of mindfulness practice and art-based therapy is found to be moderately effective for reducing mental exhaustion among healthcare workers who are prone to burnout due to the often-overwhelming stress of caring for dying patients. This result is in line with the comprehensive meta-analysis conducted by Khory et al. (2013), who have identified strong evidence to support the efficacy of mindfulness-based therapies in reducing anxiety, depression, and stress among clinical populations with consolidated effect sizes ranging from small to medium in pre-post comparison and waitlist-control studies. The result is also aligned with the burgeoning body of research that has reported creative self-expression through art making and art-based narratives as an important vehicle for burnout reduction and wellness promotion among healthcare workers (Huet and Holttum, 2016; Huet, 2017; Tjasink and Soosaipillai, 2019; Kaimal et al., 2020). Of note, treatment gains in reduced mental exhaustion were not only maintained but also enhanced by a 5-folds increase in effect size at 12-weeks follow-up. These results highlight a robust maintenance effect of MCAT, where participants continued to reap intervention benefits long after treatment completion, and are reflective of the sustained efficacy of a multimodal framework that augment mindfulness practice, expressive art making, and psychoeducation.

Second, results from this study show that MCAT was highly effective in promoting overall emotional regulation and nonreactivity to intrusive thoughts among healthcare workers who are constantly exposed to immensely emotionally charged end-of-life caregiving encounters. Treatment gains for increased emotional regulation were not only maintained but also enhanced by a 2-folds increase in effect size at 12-weeks follow-up, again accentuating a strong maintenance effect of the intervention. Moreover, new robust benefits including participants’ ability to observe their internal and external experiences including perceptions, feelings, and thoughts; to describe and label their feelings, sensations, and experiences with words; and to experience life with greater mindful awareness were identified at 12-weeks follow-up. These findings reveal that participants were able to obtain greater self-awareness and self-understanding not only during the treatment period but also well beyond treatment completion, as they continue to embark on a sustainable journey of self-care and personal growth amidst the daily challenges of caring and supporting the dying and the bereaved. These findings also aligned with a burgeoning of research that have identified the effectiveness of mindfulness practices in fostering emotional regulation, and especially magnetic resonance imaging studies (MRI) and functional MRI studies that show the ability of mindfulness meditations in activating brain regions that are involved in self-regulation, focused problem solving, adaptive behavior, and interoception (Boccia et al., 2015).

Third, the study findings reveal that MCAT was moderately effective in fostering positive attitudes toward death among healthcare workers who are faced with mortality on a day-to-day basis, including approach acceptance to death and the belief in the prospect of an afterlife where the deceased can be reunited with loved ones. While these impacts were not maintained at 12-weeks follow-up, possibly due to participants being continuously affected by their patients’ suffering and mortality, MCAT had nonetheless provided an invaluable opportunity for healthcare workers to explore and process their feelings of grief and loss in a supportive team-based environment. Such reflective opportunities are hard to come by in conventional healthcare workplace environments, if at all. In contrast, research has consistently found that palliative care professionals do not receive adequate support in dealing with the trauma and empathy fatigue resulting from deaths of patients (Kubler-Ross, 1970; Melvin, 2015; Ho, 2021). Empathy fatigue can significantly impair ones’ ability to practice their craft competently and ethically as their own wounds are continually revisited by their patients’ chronic illness, disability, mortality, and loss (Stebnicki, 2008), resulting in extraordinary stress, emotional and physical exhaustions, numbness, disengagement from patients, and the inability to continue to provide quality care. By offering an open and supportive platform to reflect and discuss their stories and experiences of grief, of which are often profound and ineffable through words but are now made accessible and articulatable through self-expressive art-based narratives, MCAT was able to create a safe space for healthcare workers to heal, to mend the wounds of their personal lives that are touched by their caregiving experiences, and to bring closure to the losses that they have encountered in their professional lives. This newly created platform and the intervention benefits that it brings can potentially be sustained through booster sessions and the establishment of regular ritualistic activities at the workplace, all of which can serve to address the collective grief experienced by the healthcare team while replenishing their empathic capacity to better cope with fatigue and burnout.

Finally, MCAT was found to have the ability to generate new and robust treatment benefits after intervention completion at 12-weeks follow-up among healthcare workers, including the cultivation of self-compassion, a deepened sense of common humanity, and elevated quality of life. While these findings are in accordance with those reported separately in research on mindful self-compassion and art-based therapy (Germer and Neff, 2013; Hass-Cohen and Findlay, 2015), MCAT removes the boundaries of intervention to form a more holistic and complete therapeutic modality that can lead to long-term benefits. Under the MCAT framework, participants can attain greater self-understanding through deeply reflecting on their past and current experiences, discover self-kindness through creative expression and articulation of their thoughts and emotions, and experience affirmation, personal growth, and collective healing by sharing their stories with supportive others in an empathic environment. All these therapeutic processes are fused to form new insights and sustainable pathways for cognitive appraisal, meaning-making, emotional regulation, self-care behaviors, and collegial support that gear toward greater mental wellbeing. This integrative mechanism further empowers narrative identity processing (Pals, 2006) for healthy personality development and positive self-transformation with difficult life experiences (McAdams, 2011).

Distinctly, the whole of MCAT is greater than the sum of its parts. Healthcare workers are not only invited to reflect on their experiences or express their minds, but also are supported through a scaffolding of intricately curated therapeutic activities that inspire continuous self-discoveries and embodied reflexive practices (Schön, 1983; Thompson and Thompson, 2008), those that can be integrated into one’s way of life and endure the test of time. Reflexivity infused with self-care capacity drives cognitive, emotional, behavioral, and relational transformations for developing sustainable resilience, marked not only by the ability to recover from and develop resistance to stressful events, but also the reconfiguration of one’s beliefs and value system to adapt and possibility withstand future adversities (Lepore and Revenson, 2006).

### Limitations and Future Research

Despite the many promising findings generated from this RCT, a few caveats must be noted. First, this is a pilot study with a small participant sample recruited from a single healthcare institution. This study scope may have influenced the implementation and outcome of MCAT, and future research could expand the sample size and study sites to include a variety of healthcare institutions, such as acute and community hospitals, in- and out-patient hospices, as well as other residential care facilities. Second, in spite of the numerous significant positive impacts that MCAT was able to generate, results revealed that treatment group participants only experienced marginally significant improvements in the primary outcome of resilience as compared to waitlist control immediately post-intervention. This finding, or lack thereof, may be due to the way in which resilience is constructed within the ER-11 measure, which comprises active engagement with the world, performance under stress, and problem-solving repertoire. This constitution does not appear to align with how resilience is being experienced by MCAT participants, which involved cognitive reappraisal, meaning-making, acceptance, and self-kindness. Future research may consider a more fitting measurement of resilience. Third, the current implementation of MCAT is manpower intensive and requires physical in-person facilitation. With the ongoing and unrelenting global public health crisis that has imposed persistent and restrictive physical distancing measures, the popularization of telemedicine and virtual social services is inevitable and may well stay as part of the new COVID-19 normal. Thus, future renditions of MCAT need to consider a digital adaption to improve accessibility, equity, and inclusion. Finally, this study is conducted in Singapore, a multiracial, and multicultural society with distinctive variants of Asian culture and languages. Hence, future research could expand the program to different socio-cultural settings to examine MCAT’s applicability and effectiveness among more diverse ethnic groups and population cohorts, including other types of caregivers, such as family careers. An ongoing study is being conducted to develop and examine a modified version of MCAT for dementia care (i.e., MCAT-DC). Concisely, a waitlist RCT design has been adopted to assess the efficacy of a 4-weekly, 10-hours, standardized, and group-based intervention for reducing caregiving stress, perceived burden, and psychological distress, while improving resilience, hope, spiritual wellbeing, and quality of life among a sample of 104 dementia family caregivers. MCAT-DC is delivered through a hybrid physical and virtual format using Zoom technology with real time programming. Future research needs to carefully consider the implementation science and target populations for enhancing acceptability and scalability in a post-pandemic world.

## Conclusion

Protecting the mental health of healthcare workers is an urgent global public health priority as called on by the World Health Organization and major healthcare institutions around the globe (World Health Organization, 2016; Søvold et al., 2021). Particularly, those immersed in palliative and end-of-life caregiving are prone to immense levels of work-related stress and alarming burnout rates. As many healthcare workers suffer in silence with poor mental health, a trickledown effect could prove detrimental to the quality, safety, and integrity of patient care. There is currently a dearth of empirically informed and clinically proven mental health intervention for this vulnerable population group. Findings from this study have revealed MCAT’s robust clinical potential to support and improve healthcare workers’ mental health through an innovative, holistic, and one-of-its-kind multimodal therapeutic framework. MCAT integrates the reflective power of mindfulness meditation with the expressive power of art-based therapy for reducing burnout, building resilience, nurturing compassion, fostering collegial support, and ultimately promoting holistic wellness. MCAT’s clinical framework is standardized, well-defined, and clearly operationalized, of which can be easily applied to and adopted in different caregiving contexts for empowering mental health self-care among diverse cohorts of caregivers. This study has generated new knowledge contributing to the advancement of theories and practices in caregiver support, mental health research, mindfulness modalities, art-based interventions, and integrative psychotherapies.

## Data Availability Statement

The original contributions presented in the study are included in the article/supplementary material, and further inquiries can be directed to the corresponding author.

## Ethics Statement

The studies involving human participants were reviewed and approved by the NTU Institutional Review Board (IRB). The patients/participants provided their written informed consent to participate in this study.

## Author Contributions

AH, GT-H, and JP conceptualized and designed the study. AH obtained the funding, project supervision, and drafted the manuscript. GT-H, TN, GO, PC, and DD was involved in the coordination and implementation of the research study, as well as drafting of the manuscript. AH and GO delivered the MCAT intervention. TN conducted the statistical analysis. All authors contributed to data interpretation, as well as the writing and revision of the manuscript.

## Funding

The MCAT study (grant no. M4081570.100) is funded by the Nanyang Technological University (NTU) Start-Up Grant, and the MCAT-DC study (grant ref. ARISE/2017/23) is funded by the ARISE Strategic Initiatives Fund of the Ageing Research Institute for Society and Education at NTU.

## Conflict of Interest

The authors declare that the research was conducted in the absence of any commercial or financial relationships that could be construed as a potential conflict of interest.

## Publisher’s Note

All claims expressed in this article are solely those of the authors and do not necessarily represent those of their affiliated organizations, or those of the publisher, the editors and the reviewers. Any product that may be evaluated in this article, or claim that may be made by its manufacturer, is not guaranteed or endorsed by the publisher.
